# Neurogenesis paradoxically decreases both pattern separation and memory interference

**DOI:** 10.3389/fnsys.2015.00136

**Published:** 2015-10-06

**Authors:** Rory Finnegan, Suzanna Becker

**Affiliations:** ^1^Neurotechnology and Neuroplasticity Lab, McMaster Integrative Neuroscience Discovery & Study, McMaster UniversityHamilton, ON, Canada; ^2^Neurotechnology and Neuroplasticity Lab, Department of Psychology, Neuroscience and Behaviour, McMaster UniversityHamilton, ON, Canada

**Keywords:** neurogenesis, dentate gyrus, sparse coding, computational modeling, restricted Boltzmann machines

## Abstract

The hippocampus has been the focus of memory research for decades. While the functional role of this structure is not fully understood, it is widely recognized as being vital for rapid yet accurate encoding and retrieval of associative memories. Since the discovery of adult hippocampal neurogenesis in the dentate gyrus by Altman and Das in the 1960's, many theories and models have been put forward to explain the functional role it plays in learning and memory. These models postulate different ways in which new neurons are introduced into the dentate gyrus and their functional importance for learning and memory. Few if any previous models have incorporated the unique properties of young adult-born dentate granule cells and the developmental trajectory. In this paper, we propose a novel computational model of the dentate gyrus that incorporates the developmental trajectory of the adult-born dentate granule cells, including changes in synaptic plasticity, connectivity, excitability and lateral inhibition, using a modified version of the Restricted Boltzmann machine. Our results show superior performance on memory reconstruction tasks for both recent and distally learned items, when the unique characteristics of young dentate granule cells are taken into account. Even though the hyperexcitability of the young neurons generates more overlapping neural codes, reducing pattern separation, the unique properties of the young neurons nonetheless contribute to reducing retroactive and proactive interference, at both short and long time scales. The sparse connectivity is particularly important for generating distinct memory traces for highly overlapping patterns that are learned within the same context.

## 1. Introduction

The role of the hippocampus in memory has been a subject of endless fascination for many decades. It is widely recognized that the hippocampus is crucial for rapid, accurate encoding and retrieval of associative memories. However, the neural mechanisms underlying these complex operations are still relatively poorly understood. Marr's theory of archicortex (Marr, 1971) was highly influential in setting the stage for subsequent computational theories of hippocampal function. At the core of his theory was the proposal that an associative memory system requires an initial coding stage followed by a subsequent processing stage that performs associative retrieval. Subsequent modelers refined Marr's ideas and further suggested that these functions of coding and retrieval map onto the known anatomical and physiological properties of the dentate gyrus and CA3 region, respectively (McNaughton and Morris, 1987; Treves and Rolls, 1992; O'Reilly and McClelland, 1994; McClelland et al., 1995; Myers and Scharfman, 2009). These models incorporate an important characteristic of the mature dentate granule cells: they are heavily regulated by feedback inhibition, resulting in extremely sparse firing and high functional selectivity (Jung and McNaughton, 1993; Chawla et al., 2005). Computer simulations demonstrate that the DG is thereby able to improve its capacity for storing overlapping memory traces by generating less overlapping neural codes, a process that has come to be known as pattern separation (Rolls, 1987; O'Reilly and McClelland, 1994; Rolls and Treves, 1998).

The discovery of adult hippocampal neurogenesis (AHN), first in rodents (Altman and Das, 1965, 1967) and subsequently in a wide range of mammalian species including humans (Eriksson et al., 1998), has forced theorists to reconsider the computational functions of the dentate gyrus. Several computational models incorporating neurogenesis have been put forward. These models postulate a range of functional roles for neurogenesis, including mitigating interference (Chambers et al., 2004; Becker, 2005; Wiskott et al., 2006; Becker et al., 2009; Cuneo et al., 2012), temporal association of items in memory (Aimone et al., 2006, 2009), and clearance of remote hippocampal memories (Chambers et al., 2004; Deisseroth et al., 2004; Weisz and Argibay, 2009, 2012). While these different theories are not necessarily incompatible with one another, they make different predictions regarding the effect of temporal spacing.

When similar items are spaced closely in time, some models predict that neurogenesis should increase pattern integration (Aimone et al., 2006, 2009). By the same token, the reverse should be true of animals with reduced neurogenesis: they should exhibit impaired pattern integration, and therefore, enhanced pattern separation for closely spaced items. Thus, factors that suppress neurogenesis such stress and irradiation (Gould et al., 1998; Wojtowicz, 2006) should impair pattern integration, resulting in *superior* abilities to distinguish similar items that are learned within the same time period. However, the opposite has been observed empirically. Rodents with reduced neurogenesis are impaired at spatial discriminations for closely spaced locations that are learned within the same session (Clelland et al., 2009), while rodents with running-induced elevated neurogenesis show enhanced performance on spatial tests of pattern separation (Creer et al., 2010). Consistent with these data, humans who have undergone several weeks of aerobic exercise training show superior performance on a within-session behavioral test of pattern separation while those with elevated stress and depression scores show a deficit on the same task (Déry et al., 2013).

When similar items are spaced widely in time, different predictions can be made regarding the fate of the item in remote memory vs. the newly learned item. Most or all computational theories agree that neurogenesis should facilitate the encoding of new items, protecting against proactive interference from previously learned information. Empirical data support this notion. For example, animals with intact levels of neurogenesis are able to learn to discriminate olfactory odor pairs that overlap with pairs learned several days ago, whereas irradiated animals with reduced neurogenesis show greater proactive interference on this task (Luu et al., 2012). On the other hand, opposing predictions arise regarding the influence of neurogenesis on remote memories. Some theories predict that neurogenesis should promote clearance of remote memories (Chambers et al., 2004; Deisseroth et al., 2004; Weisz and Argibay, 2009, 2012). Other theories make the opposite prediction, that intact neurogenesis levels should protect against retroactive interference of new learning on remote memories (Becker, 2005; Becker et al., 2009). Consistent with the latter prediction, when animals with reduced neurogenesis learn overlapping visual discriminations in different sessions spaced several days apart, the more recently learned discrimination disrupts the retrieval of the earlier memory (Winocur et al., 2012). These data support a role for neurogenesis in minimizing retroactive interference between remote and recent memories. However, it is possible that neurogenesis plays dual roles in remote memory, protecting some hippocampal memories from interference while causing other memories to decay.

Among existing computational dentate gyrus models, those that incorporate neurogenesis typically do so by either replacing existing neurons by re-randomizing their weights (Chambers et al., 2004; Becker, 2005) or introducing new neurons with random weights (Weisz and Argibay, 2009, 2012). Several additional models have looked at how regulation of neurogenesis can impact learning and plasticity by simulating dynamically regulated neural turnover and replacement. (Deisseroth et al., 2004; Crick and Miranker, 2006; Chambers and Conroy, 2007). Studies by Butz and colleagues even include a model of synaptogenesis, providing a framework for how neurogenesis regulation impacts synaptic rewiring and plasticity over varying time periods (Lehmann et al., 2005; Butz et al., 2006, 2008). However, none of these models encode the unique functional properties of young DGCs themselves into their learning rules.

How is it that AHN can contribute to improved memory and reduced interference when similar items are learned within a single session as well as when items are learned across temporal separations of days or weeks? The present study set out to investigate whether a single computational model of hippocampal coding could accommodate the role played by neurogenesis across this wide range of time scales. We propose that the functional properties of a heterogeneous ensemble of young and mature DGCs contributes to this improved memory and reduced interference among similar items. The heterogeneity of the functional properties for DGCs map closely to the developmental trajectory of adult-generated neurons, as such, our model attempts to take this trajectory into account during learning (Wang et al., 2000; McAvoy et al., 2015). In most if not all previous DG models, these characteristics have been ignored. It is known that young adult-generated neurons in the DG are more plastic, have less lateral inhibition, sparser connectivity and are more broadly tuned than their mature counter-parts. All of these may effect how young DGCs learn in relation to the existing networks of mature DGCs (Snyder et al., 2001; Schmidt-Hieber et al., 2004; Marin-Burgín et al., 2012; Dieni et al., 2013; Piatti et al., 2013; Temprana et al., 2015).

In the model described here, the maturational trajectory of adult born DGCs will be loosely based on data from the mouse, for DGCs from the third week of maturation onward. It is at about age 3–4 weeks that adult born DGCs have established synaptic afferent and efferent connections and are able to fire action potentials (Zhao et al., 2006). At this point, the young neurons still have decreased membrane resistance and elevated resting potentials, making them more excitable (Snyder et al., 2001; Schmidt-Hieber et al., 2004). Moreover, the young neurons are more sparsely connected to their perforant path inputs from the entorhinal cortex relative to mature DGCs (Piatti et al., 2013). From weeks 5 through eight the young neurons undergo a gradual decline in synaptic plasticity and are increasingly regulated by feedback inhibition (Temprana et al., 2015). By the eighth week the physiological properties of the adult-generated DGCs are largely indistinguishable from that of existing mature DGCs (Piatti et al., 2013; Temprana et al., 2015).

In this paper, we propose a novel computational model of the dentate gyrus incorporating the developmental trajectory of adult-born DGCs, using a modified version of the Restricted Boltzmann machine (RBM) to model the neural circuitry and learning equations of DGCs. As will be discussed later, an RBM is a type of neural network model consisting of 1 layer of visible and 1 layer of hidden units with each visible unit connected reciprocally to each other hidden unit. In our model, a single RBM (not stacked RBMs) will represent the EC input and DGCs with its visible and hidden units, respectively. As the model DGCs undergo development, they become progressively less plastic, more sparse in their firing, and more densely connected to their entorhinal inputs. We demonstrate how these properties can explain the importance of adult-generated DGCs at both short and long time scales.

## 2. Methods

In this section, we propose a novel approach to expressing neurogenesis in an artificial neural network model of the DG. While several replacement and additive models of neurogenesis have looked at how new neurons affect learning (e.g., Becker, 2005; Weisz and Argibay, 2009), few if any models have considered the full range of unique properties of AHN including the developmental trajectory of of adult-generated neurons: changes in plasticity, connectivity, excitability, and survival vs. apoptosis. The primary contribution of this work is to provide a computational framework within which all of these factors can be manipulated, differentiating the role of young vs. mature DGCs in memory, and the progression from one to the other. In the computational model described here we use the Restricted Boltzmann Machine (RBM) (Smolensky, 1986; Freund and Haussler, 1992; Hinton, 2002) architecture and learning procedure. RBMs are a type of generative, associative neural network model commonly used in deep learning applications (see e.g., Hinton and Osindero, 2006; Nair and Hinton, 2009). Our approach to expressing the neural trajectory of young DGCs in an RBM is by incorporating additional constraints into the learning equation, such as a dynamic learning rate and sparsity penalties. It is important to note that these are not limited to RBMs and could easily be applied to other types of neural network models (e.g., multilayer perceptrons, autoencoders, recurrent neural networks, etc.), however, there are several advantages to RBMs that will be discussed later in the discussion.

### 2.1. Restricted Boltzmann machines

A Restricted Boltzmann Machine (RBM) is a type of artificial neural network model with a simple architecture and Hebbian learning equations. The architecture of an RBM includes a set of visible and hidden units or nodes. In our model the visible units will simulate the input from the EC and the hidden units represent the DGCs. All visible nodes are fully, reciprocally connected with all hidden nodes. In the field of computer science this is referred to as a bipartite graph. Importantly, unlike the original Boltzmann machine, an RBM has no within-layer connections, making the model more tractable. The synaptic connection strengths, hereafter referred to as weights, can be described by an *N* by *M* matrix, where *N* is the number of visible units and *M* is the number hidden units. As in most artificial neural network algorithms, learning is expressed via modification of this weight matrix, according to a specific learning rule.

A Boltzmann machine learns a set of weights so as to form a probabilistic, generative model of the training data. The RBM is trained via a more tractable approximation using the contrastive divergence (CD) learning procedure (Hinton, 2002; Carreira-Perpinan and Hinton, 2005). The CD learning rule is provided in Equation (1). This equation includes positive and negative Hebbian learning terms. To obtain the visible and hidden unit states for the positive and negative terms in the learning rule, a procedure called brief Gibbs sampling is used, as detailed below.

(1) $$\Delta W_{ij}=\epsilon ({\left(v_{i}h_{j}\right)}_{data}-{\left(v_{i}h_{j}\right)}_{recon})$$

where *v*_*data*_ is the input vector and *h*_*data*_ is the data-driven hidden state generated by clamping the states of the visible units to *v*_*data*_ and sampling the hidden units' states according to Equation (2). *v*_*recon*_ is a reconstruction of the input vector generated by clamping the states of the hidden units to the data-driven pattern *h*_*data*_ and sampling the states of the visible units according to Equation (3). *h*_*recon*_ is then created in the same way as *h*_*data*_, but by clamping the visible units' states to *v*_*recon*_. In Equations (2, 3) below *a*_*i*_ and *b*_*i*_ represent biases which provide a mechanism for shifting the output of the sigmoid activation function, similar to thresholds in other neural network models.

(2) $$p(h_{j}=1|v)=\sigma (b_{j}+\sum_{i}v_{i}w_{ij})$$

(3) $$p(v_{i}=1|h)=\sigma (a_{i}+\sum_{j}h_{j}w_{ij})$$

As can be seen from the CD learning (Equation 1), the positive Hebbian term associates data-driven input and hidden state vectors, while the negative Hebbian term tries to “unlearn” the association between the corresponding reconstructed visible and hidden state vectors. Theoretically, the learning procedure should converge when its internal reconstructions of the training patterns exactly match the corresponding data-driven states. In general, an RMB model's reconstructions of the training patterns are obtained by alternatingly sampling nearby hidden and visible unit states using the model's bottom-up and top-down weights, respectively. In the simulations reported here, we applied this brief Gibbs sampling procedure for just one iteration. Performance of this model can be improved further by performing multiple steps of brief Gibbs sampling (Hinton, 2012). The Boltzmann machine learning procedure is normally performed repeatedly for many iterations through the training set. In contrast, here we simulated just one exposure to each training pattern.

### 2.2. Sparsity

In our simulations of neurogenesis, we take into consideration both sparse coding and sparse connectivity. Sparse coding means that very few strongly activated neurons respond to a given event. This helps to improve pattern separation as it minimizes the probability of overlap in the model's internal representation of highly similar input patterns. As noted above, extreme sparse coding is observed in mature DG granule cells, but not in less mature adult-generated neurons. In our model we simulate sparse coding by incorporating a sparsity cost constraint into the learning objective. Our sparse coding constraint is the average squared difference between each hidden unit's average activation and it's target probability of activation (Nair and Hinton, 2009). By taking the derivative of this cost term with respect to the weights, we obtain an added component to the learning equation that adjusts the weights so as to penalize units whose activation deviates from a target level of sparseness. The relative importance of this sparse coding term increases with the age of the neurons, to simulate the increased degree of connectivity with inhibitory interneurons of mature DGCs. In the updated learning equation below *q* is the mean of our sampled hidden activation from Equation (2) and *p* is our target activation probability.

(4) $$\Delta W_{ij}=\epsilon ({\left(v_{i}h_{j}\right)}_{data}-{\left(v_{i}h_{j}\right)}_{recon})-cost*(q-p)$$

Sparse connectivity describes the level of interconnectedness between the visible and hidden layers. As mentioned earlier, the degree of inter-connectivity is another property that changes as the young DGCs mature.

We simulate the maturational evolution of increased sparse coding and decreased sparse connectivity as follows. In the case of sparse coding we vary the weight on the sparsity cost for each hidden unit so that it is smaller for young neurons and larger for their mature counterparts. To impose a sparse connectivity constraint, a binary matrix is used as a connectivity mask for the weight matrix. As the hidden units mature, the number of non-zero visible-to-hidden connections in the connectivity matrix for that hidden unit is increased probabilistically. At the end of each weight update the weight matrix is multiplied by this connectivity mask in order to maintain the “disconnected” links to have weights of zero.

### 2.3. Neuron growth

Our model makes the assumption that young neurons are more plastic, have less lateral inhibition (simulated via our sparse coding cost) and are more sparsely connected than their mature counterparts (Wang et al., 2000; Schmidt-Hieber et al., 2004; Oswald and Reyes, 2008; Marin-Burgín et al., 2012). For simplicity, we assume that each of these characteristics follows a temporal growth curve that can be described with some permutation of the Gompertz function (Gompertz, 1832). The Gompertz function has been used to model growth in a variety of applications ranging from modeling bacterial growth in biology to product demand in economics (Zwietering et al., 1990; Towhidul et al., 2002).

(5) $$g(t)=e^{-e^{-st}}$$

**Figure 1:**
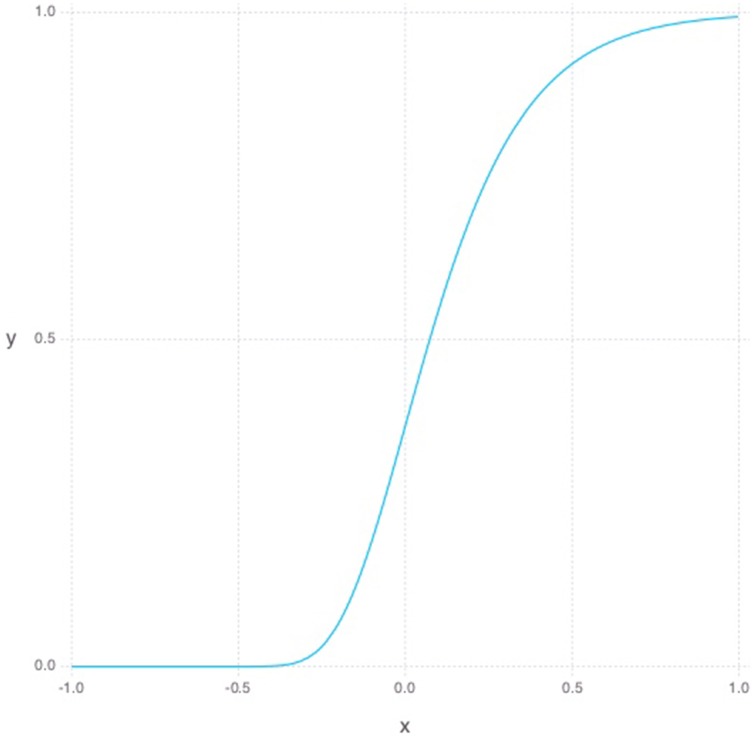
**Gompertz function where *s* is set to 5 and *t* is between −1 and 1**.

The Gompertz function in Equation (5) defines a sigmoid-like growth curve, where *t* describes the time step and *s* describes the shape or steepness of the function as can be seen in Figure 1. For our purposes, *t* is bounded between -1 and 1 and the *s* is always set to 5. To model young DGC growth characteristics in the RBM, each hidden neuron has its own set of parameters defining its current learning rate and sparsity constraints. Additionally, each hidden unit has a time parameter representing its age. At each simulated unit time interval, the age of a hidden unit is increased, and its constraint parameters are updated as follows. The learning rate, which can be thought of as a neuron's plasticity level, is defined as 1−*g*(*t*) normalized to lie between 0.1 and 0.3. Inversely, our sparsity cost can simply be taken from *g*(*t*) and normalized to lie between 0 and our initial sparsity cost of 0.9. Given these variable properties, the learning rule can be redefined as

(6) $$\Delta W_{ij}=\epsilon ({\left(v_{i}h_{j}\right)}_{data}-{\left(v_{i}h_{j}\right)}_{recon}))-(\lambda \;*\;W_{ij})-cost\;*\;(q-p)$$

where the learning rate ϵ, weight decay λ and sparsity cost terms are now each weighted by dynamically changing vectors of values rather than static hyper-parameters.

### 2.4. Neural turnover

It is difficult to estimate the rate at which adult-generated neurons undergo apoptosis vs. survival and maturation into adult DGCs. These processes are governed by many factors (see, e.g., Hutchins and Barger, 1998; Cameron and McKay, 2001; Cecchi et al., 2001; Elmore, 2007) and are not completely understood. Generally apoptosis among healthy neurons tends to be activity and age dependent (Hutchins and Barger, 1998; Cecchi et al., 2001) and a significant number of new DGCs survive to adult hood (Cameron and McKay, 2001). Using these observations, we formulate a rule for determining whether a given neuron will survive or undergo apoptosis based on its age, specificity and average synaptic strength. To assess stimulus specificity, we calculate the standard deviation of each hidden unit's incoming weights, a quantity we refer to hereafter as its “differentiation.” The justification is that hidden units with equal weight to all visible units will be less effective at differentiating different input patterns. Similarly, to assess synaptic strength we calculate the average absolute value of the those incoming weights. Combining the differentiation and synaptic strength penalty terms, we are penalizing hidden units with incoming weights that are all very similar and close to zero. We rank each hidden neuron based on a weighted average of its synaptic strength, differentiation and age with the equation given below. Neurons within the lowest 5% of this ranking undergo simulated apoptosis by having their age reset to 0 and weights reset to random initial values (or set to 0 in the case of bias weights).

(7) $$Z_{i}\text{​}=\text{​}(\alpha \;*\;Strength_{i}+\beta \;*\;Differentiation_{i}+\gamma \;*\;Age_{i})/(\alpha +\beta +\gamma )$$

where

- *Strength*_*i*_ is the average of the weights from all visible units to a given hidden unit *i*.
- *Differentiation*_*i*_ is the standard deviation of the visible weights to hidden unit *i*
- *Age*_*i*_ is our recorded age for the hidden unit *i*
- α, β, and γ are coefficients for modifying the relative importance of the *Strength, Differentiation*, and *Age* terms. For our simulations these are set to 0.2, 0.65, and 0.15, respectively.

### 2.5. Experiments

All models simulated in the experiments reported here used CD with 1 step Gibbs sampling on a single layer RBM as described above. A learning rate of 0.1 was used for all non-neurogenesis models and a value between 0.1 and 0.3 was used for all neurogenesis models. For all sparse coding models the expected probability of activation for each hidden unit (representing the target sparseness of mature DGCs) was set to 0.05. This is a very conservative constraint as previous models and empirical studies have this set at around an order of magnitude lower, 0.004 or 0.4% (Barnes et al., 1990; Jung and McNaughton, 1993). All models had 200 visible units and 1000 hidden units in order to roughly match the relative numbers of EC and DG neurons, respectively observed in rodents, as in previous models (O'Reilly and McClelland, 1994). For all experiments, each model was trained on mini-batches of 5 training patterns at a time, 1 sample from each parent class as described below. In order to simulate rapid one-shot learning, only 1 iteration through the training set was taken. Similar to Orielly and McClelland (O'Reilly and McClelland, 1994), we set the expected probability of activation of each unit in the training and test patterns (representing the activation level of each EC input unit) to be 0.1.

Each simulated model was trained on a set of binary patterns representing input from the entorhinal cortex. These patterns were randomly generated, with 10 percent of the elements of each pattern being active (set to 1.0) and the remainder inactive (set to 0.0). The patterns were created as random variations on a base set of prototypes, so as to create patterns that had varying degrees of similarity. Initially, five binary seed patterns were created, representing prototype patterns from 5 different classes. For each of these classes, 10 additional overlapping prototypes were generated by randomly resetting 20% percent of the original pattern. From these 55 prototypes (representing 5 classes and 11 subclasses per class), 1200 patterns were generated and partitioned into 1000 training patterns and 200 test patterns. Each of these patterns were created by randomly resetting another 5% of the elements in one of the subclass patterns.

While the training and testing scenarios varied between experiments, our evaluation of performance remained the same. A test pattern was presented to the model and the Hamming distance between the input pattern and the model's reconstruction of that test pattern was calculated using Equation (8). From there the percent match was calculated using Equation (10), where *l* is the length of *V*_*data*_ / *V*_*recon*_. This metric serves as an approximation of the formal log-likelihood cost function for the Boltzmann model, however, other approximations such as brief gibbs sampling and small mini-batches are inherent to the RBM model.

(8) $$D(V_{data},V_{recon})\;=\sum_{i=1}^{n}|(V_{data_{i}}-V_{recon_{i}})|$$

(9) $$M(V_{data},V_{recon})\;=1-(D(V_{data},V_{recon})/l)$$

Before introducing neurogenesis into the models, in simulation 1, we evaluated the contribution of sparse coding to associative memory in the DG model. Thus, we compared the accuracy of the sparse coding RBM with the base RBM lacking a sparse coding constraint. We hypothesized that the sparse coding RBM would perform better, particularly for encoding highly similar patterns. We evaluated this and all other models on both proactive and retroactive interference. Learning a pattern that is highly similar to the model previously learned is a source of proactive interference, potentially making it more difficult to encode the current pattern. Additionally, learning the current pattern could interfere retroactively with the model's ability to retrieve a previously learned overlapping pattern. Thus, each model was trained on groups of patterns, consisting of all training patterns from 5 of the 55 prototypes (90 patterns for a training set of 1000), one from each class, and immediately tested with the corresponding test patterns on its accuracy at reconstructing these patterns. As mentioned above these patterns were presented to the model in mini-batches of 5 (1 example per class), and the training and test patterns had noise added to them from their prototypes by randomly resetting 5% of the elements. It was then trained on another group of 90 patterns with one prototype selected from each class, with each successive group of 90 patterns overlapping with previously learned patterns. After learning the entire set of 1000 patterns consisting of 11 groups of 90, the model was finally tested on its ability to reconstruct all test patterns from all previously learned groups to test retroactive interference.

In Simulation 2, the sparsely coded RBM with neurogenesis, with and without sparse connectivity, was compared to the sparse RBM. We were particularly interested in how the neurogenesis model would perform at encoding and recognizing similar patterns when they were encountered within the same learning session vs. across different learning sessions spaced more widely in time. We therefore compared the performance of the various models across 2 conditions: (1) same-session testing in which the neurogenesis models had no neural turnover or growth, (2) multi-session testing which had both neural growth and neural turnover. The same-session testing condition was created with no simulated passage of time after training on each successive group of 90 patterns. In contrast, for multi-session training conditions the passage of time between training on blocks of 90 patterns were simulated by incrementing the neuron age parameter for all hidden units after each group of 90 patterns. As discussed previously neural growth was simulated by incrementing the age parameter and recomputing the learning rate and sparsity cost using the Gompertz function for each hidden unit. Similarly, to simulate neural turnover, we ranked the performance of each hidden unit based on the weighted average of the synaptic strength, differentiation, and age as described earlier, and reinitialized the lowest 5%. Both neural turnover and growth were performed between sessions (or groups of 90 patterns) when we incremented the age parameter of the hidden units.

Our hypothesis for same-session testing was that the neurogenesis models would perform better than the sparsely coded RBM without neurogenesis due to the presence of a few young more plastic neurons. Further, because the available pool of young excitable neurons would be constant for same-session learning, making it difficult for the model to generating distinctive traces for similar items experienced within the same context, we predicted that sparse connectivity would be particularly important for same-session learning. For multi-session testing, giving that a new pool of young neurons would be available at each learning session, we hypothesized that the neurogenesis models would perform even better then they did for same-session testing. Further, allowing some of the young neurons to mature and forcing less useful neurons to be replaced was predicted to lead to improved reconstruction accuracy with lower proactive and retroactive interference.

## 3. Results

**Figure 2:**
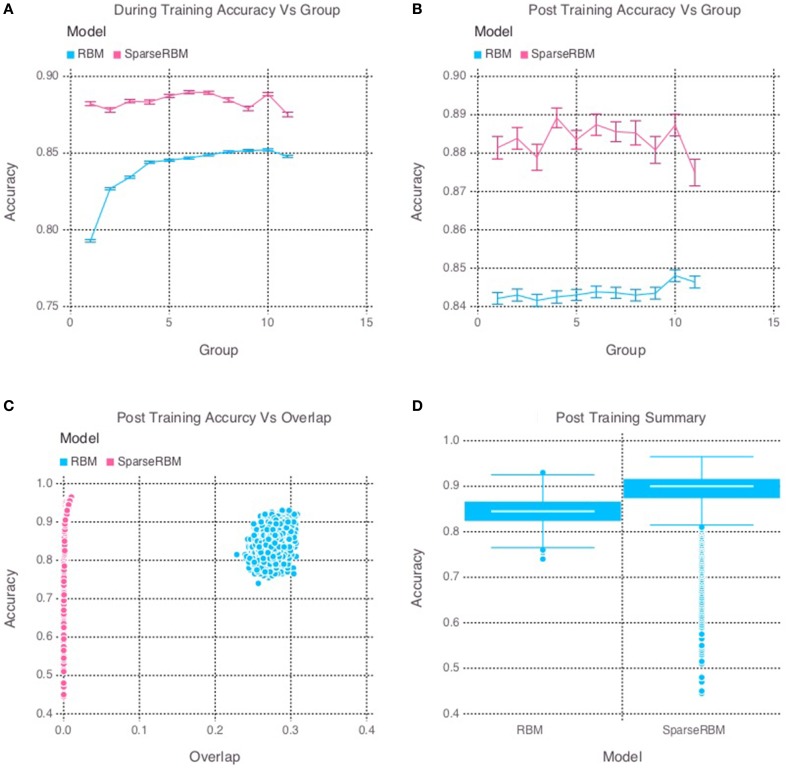
**Simulation 1: performance of the models with and without sparse coding on within-session pattern reconstruction tests**. The models were trained sequentially on 11 groups of 90 patterns, and tested on noisy versions of these training patterns after each group to test proactive interference and after all groups had completed to test retroactive interference. **(A)** Shows proactive interference for input reconstruction accuracies during training. **(B)** Shows retroactive interference for input reconstruction accuracies on each group after training to test retroactive interference. **(C)** Shows the relationship between post training reconstruction accuracy with hidden unit activation overlap. **(D)** Shows the distribution of post training accuracy over all groups.

The results from initial tests comparing the sparse coding RBM with the base RBM, show a significant improvement in overall reconstruction accuracy, as can be seen in both the during and post training tests shown in Figures 2A,B, respectively, as well in the summary graph in Figure 2D. Similarly, the sparse coding was shown to be effectively helping to increase pattern separation, as can be seen by the reduced pattern overlap of the hidden unit activations in Figure 2C. It is note worthy that the overlap for the base RBM was less than 30% and the slow increase in performance during training suggests that it was able to learn the sparse representation of the dataset to some extent, but not as quickly as its sparse counterpart.

**Figure 3:**
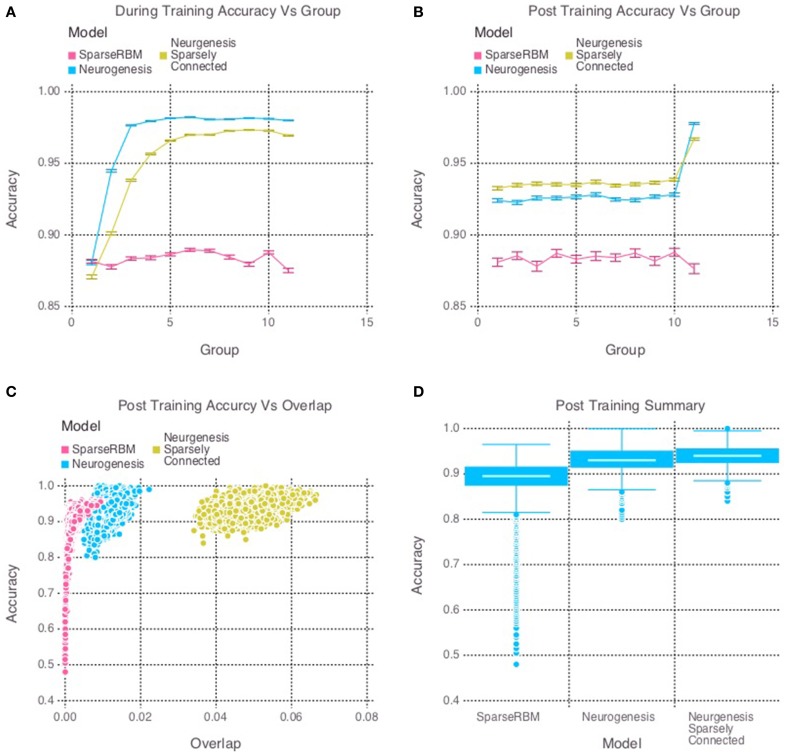
**Simulation 2: performance of the models with and without neurogenesis and sparse connectivity on within-session pattern reconstruction tests**. The models were trained sequentially on 11 groups of 90 patterns, and tested on noisy versions of these training patterns after each group to test proactive interference and after all groups had completed to test retroactive interference. **(A)** Shows proactive interference for input reconstruction accuracies during training. **(B)** Shows retroactive interference for input reconstruction accuracies on each group after training to test retroactive interference. **(C)** Shows the relationship between post training reconstruction accuracy with hidden unit activation overlap. **(D)** Shows the distribution of post training accuracy over all groups.

The same session tests showed improved accuracy for both neurogenesis models, even without neural aging or turnover. This was expected since the initial age of the hidden units were randomly selected, allowing the encoded characteristics of our young neurons to provide the necessary advantage. The sparse connectivity appears to provided a further advantage for same session testing as we can see in Figure 3D. Interestingly, Figure 3C shows that the neurogenesis models have more overlap among hidden unit activation than the normal sparse RBM, which demonstrates that the neurogenesis models are providing an opportunity to have slightly less sparse activations due to the young neurons. Another interesting pattern that can be seen in Figure 3B, which shows a kind of recency effect found in numerous memory studies (Murdock, 1962). At the same time, Figure 3A, show the neurogenesis models having reduced proactive interference. The increase in accuracy on subsequent groups of patterns suggests that the neurogenesis models may be better at distinguishing novel and common elements to each group of patterns.

**Figure 4:**
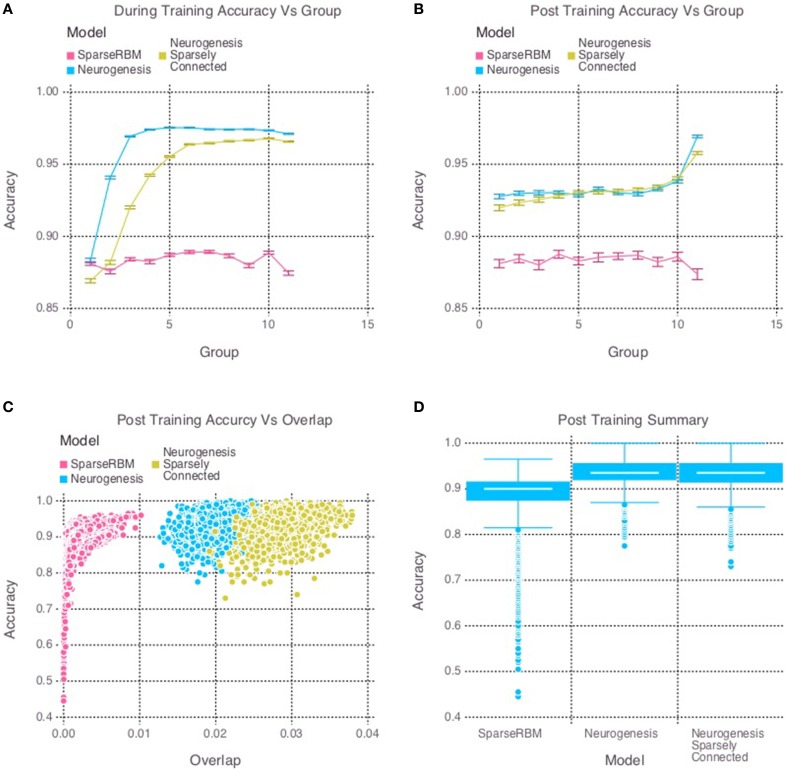
**Simulation 2: performance of the models with and without neurogenesis and sparse connectivity on across-session pattern reconstruction tests**. The models were trained sequentially on 11 groups of 90 patterns, and tested on noisy versions of these training patterns after each group to test proactive interference and after all groups had completed to test retroactive interference. **(A)** Shows proactive interference for input reconstruction accuracies during training. **(B)** Shows retroactive interference for input reconstruction accuracies on each group after training to test retroactive interference. **(C)** Shows the relationship between post training reconstruction accuracy with hidden unit activation overlap. **(D)** Shows the distribution of post training accuracy over all groups.

The multi session tests showed similar improvement as expected. Figure 4D once again shows the neurogenesis models outperforming the sparse RBM model. Once again, we can see from Figures 4A,B a recency effect and reduced proactive interference from the neurogenesis models. However, the use of neural maturation and turnover in the multi session tests provided less benefit to overall performance than expected. While the non-sparsely connected neurogenesis model did see about a 1% increase in performance from the same session tests, the sparsely connected neurogenesis model saw no improvement and did about the same as its non-sparse counterpart. Interestingly, Figure 4C shows that the increased overlap for the sparsely connected model is no longer present for our multi session tests and instead the overlap for the non-sparsely connected neurogenesis model has increased. This latter point, suggests that the sparse connectivity and neural turnover work in equilibrium with each other depending on the learning demands required.

In summary, the results from the neurogenesis tests showed an improvement over the sparse coding RBM in all cases with and without sparse connectivity. Similarly, the sparse connectivity did show better performance on the same session scenario, however, it showed no significant improvement for multisession tests. This suggests that the sparse connectivity of young neurons provides improved performance on pattern separation and completion tasks in the short-term, but provides little benefit for longer term applications. Table 1 shows the mean values and confidence intervals from the post training tests for each simulation.

**Table 1:** **Post training summary statistics for the 3 simulations**.

**Simulation**	**Models**	**Means**	**Confidence Interval**	**Significant**
**1 - SAME-SESSION**				
	RBM vs. SparseRBM	(0.844, 0.884)	(0.03, 0.054)	^*^
**2 - SAME-SESSION**				
	SparseRBM vs. Neurogenesis	(0.883, 0.938)	(0.035, 0.057)	^*^
	SparseRBM vs. Neurogenesis sparsely connected	(0.883, 0.938)	(0.04, 0.065)	^*^
	Neurogenesis vs. Neurogenesis sparsely connected	(0.93, 0.938)	(0.006, 0.01)	^*^
**2 - MULTI-SESSION**				
	SparseRBM vs. Neurogenesis	(0.883, 0.934)	(0.04, 0.06)	^*^
	SparseRBM vs. Neurogenesis sparsely connected	(0.883, 0.932)	(0.037, 0.058)	^*^
	Neurogenesis vs. Neurogenesis sparsely connected	(0.934, 0.932)	(−0.004, 0.0)	

Mean accuracies of each pair of models and 99% bootstrapped confidence intervals around the difference between means are shown;

* *'s indicate statistically significant differences (those with confidence intervals which do not include 0). The confidence intervals were generated by calculating the difference in mean performance of pairs of models across 20 repeated simulations with different randomly generated training and test sets. From these 20 repeated simulations, we generated 10,000 bootstrapped resamples, to obtain bootstrapped estimates of the distributions of the mean differences*.

## 4. Discussion and future work

The main goal of this paper was to investigate whether the unique characteristics of young adult-born DGCs during their maturation period, such as increased synaptic plasticity and reduced lateral inhibition (Schmidt-Hieber et al., 2004; Marin-Burgín et al., 2012), contribute to learning novel, highly overlapping patterns. We were particularly interested in the potential contribution of these various properties of young neurons to interference reduction when similar patterns are encountered at short vs. long time spacings.

We chose to simulate the contribution of neurogenesis to memory encoding using a Restricted Boltzmann Machine (RBM) to simulate the dentate gyrus circuitry. This was achieved by adding a set of additional constraints to the RBM learning rule to simulate the properties of young immature neurons as they evolve over time into mature granule cells. While several neural network models exist which are more biologically plausible than RBMs, the RBM has several useful properties which require little computational overhead. Unlike most other types of artificial neural network models RBMs can be stacked and trained sequentially to form deep multilayer networks without relying on back-propagation. In contrast, deep networks trained by the error back-propagation learning procedure (LeCun, 1985; Rumelhart et al., 1986) suffer from the vanishing gradient problem (Hochreiter et al., 2001). Put another way, the learning typically gets lost in the noise and converges on a very poor set of weights. Furthermore, these models are considered to be less biologically plausible due the requirement of non-local computations (Stocco et al., 2011). The RBM has the additional advantage of forming a generative model of the data. Hence, this model can generate novel input patterns from the same data distribution that it was trained on. It thereby has the potential to simulate cognitive processes such as memory reconstruction and consolidation (Kali and Dayan, 2002). RBMs have also been used in various challenging machine learning problems ranging from image and document classification, (Hinton and Osindero, 2006; Salakhutdinov and Hinton, 2010) to user rating systems (Salakhutdinov et al., 2007), but have rarely been used in modeling the nervous system. Given that our objective was to see how the variability in plasticity, lateral inhibition, and connectivity among a heterogenous pool of young and mature DGCs impacts memory and interference, the RBM satisfied our requirements. As previously mentioned, our learning rule modifications are not specific to the RBM and could easily be combined with other neural network learning rules. For example, autoencoders, multilayer perceptrons, and recursive neural networks can all use the same variability in learning rate, weight decay, and sparsity constraints based on the age of the neurons in the DG layer.

Previous modeling studies have shown that the sparse coding caused by lateral inhibition within the DG results in improved pattern separation (O'Reilly and McClelland, 1994) which is useful for distinguishing highly similar patterns. We reaffirmed this in simulation 1, where we compared the reconstruction of highly similar patterns for an RBM with and without a sparse coding constraint. Similar to previous studies, we found significantly better performance for the RBM using a sparse coding constraint.

Our main finding is that the models with a mixture of young and old neurons did not learn a neural code that maximized pattern separation, and yet they outperformed models with sparser, less overlapping codes but lacking neurogenesis. This may seem counter-intuitive in light of the findings of simulation 1: for models lacking neural turnover, those with a sparse coding constraint were superior. An alternative explanation for these results is that the degree of pattern separation achieved by the control model (sparsely coded RBM lacking neurogenesis) was so high (less than 0.05% pattern overlap in some cases; see Figure 3C) that it would be impossible for models without such a sparseness constraint on the young neurons to achieve the same degree of pattern separation. However, a closer examination of the distribution of pattern separation scores vs. model performance makes this explanation seem unlikely. The RBM has the flexibility to learn any neural code that is optimal for pattern reconstruction, ranging from a sparse code to a highly distributed code. In fact, the sparse RBM and the RBM with neurogenesis produced codes with varying degrees of pattern separation in different cases (see Figure 3C), and there was considerable overlap in the distributions of pattern separation scores for the two models. In cases where the sparse RBM achieved the highest degree of pattern separation (the bottom tail of the distribution in Figure 3C) the sparse RBM actually performed most poorly. In other cases where the sparse RBM converged to somewhat less sparse codes, performance appeared to be asymptotically approaching about 95% (the top end of the distribution in Figure 3C). On the other hand, models with neurogenesis achieved performance approaching 100%, in spite of a wide range of pattern separation scores; in some situations the neurogenesis models achieved comparable pattern separation to the sparse RBM but still produced superior performance. These results support our main conclusion that a heterogeneous model with a balance of mature more sparsely firing neurons and younger neurons with higher firing rates achieves superior pattern encoding relative to a purely sparse code. While our simulations suggest that the addition of younger, more hyperactive neurons strictly leads to reduced pattern separation, McAvoy et al. (2015) suggest that young neurons may counter this effect via potent feedback inhibition of mature granule cells. The latter mechanism could thus compensate for the increased activity in the young neuronal population by inducing greater sparsity in the mature population. The net result of this could be a homeostatic maintenance of the overall activity level in the dentate gyrus (McAvoy et al., 2015). In either case, pattern separation is obviously not a strict requirement for accurate neural coding. The more distributed code learned by the models with a pool of younger neurons seems to offer a good compromise between high pattern separation and high plasticity.

Sparse connectivity was found to be critical when the model attempted to encode similar patterns encountered within a single training session. In this case, the model would not have the opportunity to generate a set of new neurons between encoding of one similar pattern and the next, and it therefore had to rely on sparse connectivity of the young neurons to generate distinct responses to similar patterns. Across a longer temporal separation, some of the young neurons would have matured while there would be additional young, more plastic neurons available to encode successive similar patterns. Thus, these additional properties of greater plasticity and higher activation were more important for separating patterns that were encountered across longer time scales. While these results shed light on the ways in which different features of young neurons may contribute to memory, there are several limitations to our models that will need to be addressed in future work.

The current model using the RBM requires reciprocal connectivity between the input and output layers, whereas the known anatomy of the dentate gyrus does not support this architecture; dentate granule cells do not project back to the entorhinal cortex. However, in an elaborated version of this model (Becker and Hinton, 2007) that will be developed further in future work, we incorporate the reciprocal connections between the CA3 and the dentate gyrus (Myers and Scharfman, 2011), and between the CA3 and the entorhinal cortex, thus providing a natural fit of the stacked RBM architecture as described earlier to that of the hippocampal circuit. This full hippocampal circuit model will be required to explore the functional impact of young vs. mature DGCs on hippocampal learning, particularly when investigating the performance changes on memory recall (pattern completion) and sequence replay tasks. Similarly, the the generative characteristics of the RBM combined with this stacked architecture provide a method of simulating imagination and dreaming along with memory reconstruction.

The model of the young adult-born DGC maturation presented in this paper looked specifically at changes in synaptic plasticity and lateral inhibition during the cell development trajectory, however, it does not take into account temporal changes in action potential kinetics (Schmidt-Hieber et al., 2004; Marin-Burgín et al., 2012). This temporal component would be a valuable contribution for future work, particularly when modeling spatio-temporal learning and sequence replay (Karlsson and Frank, 2009).

Finally, we modeled neurogenesis and apoptosis as one operation with the simplified replacement approach. However, in future work neurogenesis and apoptosis should be treated as two independent processes for regulating the population of DGCs. We propose creating a hybrid additive and replacement model in which neurogenesis can be up or down regulated in order to better investigate the role of neurogenesis in pattern separation and completion tasks over varying time spans. This ability to up and down regulate neurogenesis could prove extremely useful in exploring the results of recent studies examining the potential role of neurogenesis in human memory at both short and long time scales. A recent study by Dery, Goldstein & Becker showed that lower stress and depression scores, which were presumed to correlate with higher neurogenesis levels, result in improved item recognition over larger time spans (2 weeks) (Déry et al., 2015).

In summary, our results suggest that the developmental trajectory of adult-born DGCs may be important in explaining the role of young neurons in interference reduction at both short and long time scales. Interestingly, even though the young neurons decrease sparseness and pattern separation, they play a critical role in mitigating both retroactive and proaction interference. Future work in this area should address the following questions: The most important are (1) What is the functional impact of DGC maturation on full Hippocampal learning tasks? (2) How do changes in the temporal dynamics of action potentials between young and mature DGCs impact these results? (3) How could this model of young vs. mature DGCs be expanded into a hybrid additive & replacement model?

### Conflict of interest statement

The authors declare that the research was conducted in the absence of any commercial or financial relationships that could be construed as a potential conflict of interest.

## References

[B1] Aimone J. B. Wiles J. Gage F. H. (2006). Potential role for adult neurogenesis in the encoding of time in new memories. Nat. Neurosci. 9, 723–727. 10.1038/nn1707 16732202

[B2] Aimone J. Wiles J. Gage F. (2009). Computational influence of adult neurogenesis on memory encoding. Neuron 61, 187–202. 10.1016/j.neuron.2008.11.026 19186162 PMC2670434

[B3] Altman J. Das G. D. (1965). Post-natal origin of microNeurons in the rat brain. Nature 207, 953–956. 5886931 10.1038/207953a0

[B4] Altman J. Das G. (1967). Postnatal neurogenesis in the guinea-pig. Nature 214, 1098–1101. 6053066 10.1038/2141098a0

[B5] Barnes C. A. McNaughton B. L. Mizumori S. J. Leonard B. W. Lin L. H. (1990). Comparison of spatial and temporal characteristics of Neuronal activity in sequential stages of hippocampal processing. Prog. Brain Res. 83, 287–300. 2392566 10.1016/s0079-6123(08)61257-1

[B6] Becker S. Hinton G. E. (2007). Caching and replay of place sequences in a Temporal Restricted Boltzmann Machine model of the hippocampus, in Cosyne (abstract only, Poster II-56). Available online at: http://www.cosyne.org/c/images/c/cf/Cosyne-poster-II-56.pdf

[B7] Becker S. Macqueen G. Wojtowicz J. W. (2009). Computational modeling and empirical studies of hippocampal neurogenesis-dependent memory: effects of interference, stress and depression. Brain Res. 1299, 45–54. 10.1016/j.brainres.2009.07.095 19651106

[B8] Becker S. (2005). A computational principle for hippocampal learning and neurogenesis. Hippocampus 15, 722–738. 10.1002/hipo.20095 15986407

[B9] Butz M. Lehmann K. Dammasch I. E. Teuchert-Noodt G. (2006). A theoretical network model to analyse neurogenesis and synaptogenesis in the dentate gyrus. Neural Netw. 19, 1490–1505. 10.1016/j.neunet.2006.07.007 17014989

[B10] Butz M. Teuchert-Noodt G. Grafen K. van Ooyen A. (2008). Inverse relationship between adult hippocampal cell proliferation and synaptic rewiring in the dentate gyrus. Hippocampus 18, 879–898. 10.1002/hipo.20445 18481284

[B11] Cameron H. A. McKay R. D. (2001). Adult neurogenesis produces a large pool of new granule cells in the dentate gyrus. J. Comp. Neurol. 435, 406–417. 10.1002/cne.1040 11406822

[B12] Carreira-Perpinan M. A. Hinton G. E. (2005). On contrastive divergence learning, in Proceedings of the Tenth International Workshop on Artificial Intelligence and Statistics, eds Cowell R. G. Ghahramani Z. (Barbados: Society for Artificial Intelligence and Statistics), 33–40.

[B13] Cecchi G. A. Petreanu L. T. Alvarez-Buylla A. Magnasco M. O. (2001). Unsupervised learning and adaptation in a model of adult neurogenesis. J. Comput. Neurosci. 11, 175–182. 10.1023/A:1012849801892 11717533

[B14] Chambers R. A. Conroy S. K. (2007). Network modeling of adult neurogenesis: shifting rates of neuronal turnover optimally gears network learning according to novelty gradient. J. Cogn. Neurosci. 19, 1–12. 10.1162/jocn.2007.19.1.1 17214558 PMC2887709

[B15] Chambers R. A. Potenza M. N. Hoffman R. E. Miranker W. (2004). Simulated apoptosis/neurogenesis regulates learning and memory capabilities of adaptive neural networks. Neuropsychopharmacology 29, 747–758. 10.1038/sj.npp.1300358 14702022

[B16] Chawla M. K. Guzowski J. F. Ramirez-Amaya V. Lipa P. Hoffman K. L. Marriott L. K.. (2005). Sparse, environmentally selective expression of arc rna in the upper blade of the rodent fascia dentata by brief spatial experience. Hippocampus 15, 579–586. 10.1002/hipo.20091 15920719

[B17] Clelland C. D. Choi M. Romberg C. Clemenson G. D. Jr. Fragniere A. Tyers P.. (2009). A functional role for adult hippocampal neurogenesis in spatial pattern separation. Science 325, 210–213. 10.1126/science.1173215 19590004 PMC2997634

[B18] Creer D. J. Romberg C. Saksida L. M. van Praag H. Bussey T. J. (2010). Running enhances spatial pattern separation in mice. Proc. Natl. Acad. Sci. U.S.A. 107, 2367–2372. 10.1073/pnas.0911725107 20133882 PMC2836679

[B19] Crick C. Miranker W. (2006). Apoptosis, neurogenesis, and information content in hebbian networks. Biol. Cybern. 94, 9–19. 10.1007/s00422-005-0026-8 16372165

[B20] Cuneo J. I. Quiroz N. H. Weisz V. I. Argibay P. F. (2012). The computational influence of neurogenesis in the processing of spatial information in the dentate gyrus. Sci. Rep. 2:735. 10.1038/srep00735 23071899 PMC3471095

[B21] Déry N. Pilgrim M. Gibala M. Gillen J. Wojtowicz J. M. Macqueen G.. (2013). Adult hippocampal neurogenesis reduces memory interference in humans: opposing effects of aerobic exercise and depression. Front. Neurosci. 7:66. 10.3389/fnins.2013.00066 23641193 PMC3639381

[B22] Déry N. Goldstein A. Becker S. (2015). A role for adult hippocampal neurogenesis at multiple time scales: a study of recent and remote memory in humans. Behav. Neurosci. 129, 435–449. 10.1037/bne0000073 26076341

[B23] Deisseroth K. Singla S. Toda H. Monje M. Palmer T. D. Malenka R. C. (2004). Excitation-neurogenesis coupling in adult neural stem/progenitor cells. Neuron 42, 535–552. 10.1016/S0896-6273(04)00266-1 15157417

[B24] Dieni C. V. Nietz A. K. Panichi R. Wadiche J. I. Overstreet-Wadiche L. (2013). Distinct determinants of sparse activation during granule cell maturation. J. Neurosci. 33, 19131–19142. 10.1523/JNEUROSCI.2289-13.2013 24305810 PMC3850038

[B25] Elmore S. (2007). Apoptosis: a review of programmed cell death. Toxicol. Pathol. 35, 495–516. 10.1080/01926230701320337 17562483 PMC2117903

[B26] Eriksson P. S. Perfilieva E. Björk-Eriksson T. Alborn A.-M. Nordborg C. Peterson D. A.. (1998). Neurogenesis in the adult human Hippocampus. Nat. Med. 4, 1313–1317. 9809557 10.1038/3305

[B27] Freund Y. Haussler D. (1992). Unsupervised learning of distributions on binary vectors using two layer networks, in Advances in Neural Information Processing Systems 4 (San Mateo, CA: Morgan Kaufmann), 912–919.

[B28] Gompertz B. (1832). On the nature of the function expressive of the law of human mortality, and on a new method of determining the value of life contingencies. Philos. Trans. R. Soc. Lond. 123, 513–585. 10.1098/rstb.2014.0379 PMC4360127 25750242

[B29] Gould E. Tanapat P. McEwen B. S. Flügge G. Fuchs E. (1998). Proliferation of granule cell precursors in the dentate gyrus of adult monkeys is diminished by stress. Proc. Natl. Acad. Sci. U.S.A. 95, 3168–3171. 9501234 10.1073/pnas.95.6.3168 PMC19713

[B30] Hinton G. E. Osindero S. (2006). A fast learning algorithm for deep belief nets. Neural Comput. 18, 1527–1554. 10.1162/neco.2006.18.7.1527 16764513

[B31] Hinton G. (2002). Training producst of experts by minimizing contrastive divergence. Neural Comput. 14, 1771–1800. 10.1162/089976602760128018 12180402

[B32] Hinton G. (2012). A practical guide to training restricted boltzmann machines, in *Neural Networks: Tricks of the Trade* Vol. 7700 *of Lecture Notes in Computer Science*, eds Montavon G. Orr G. Müller K.-R. (Berlin; Heidelberg: Springer), 599–619.

[B33] Hochreiter S. Bengio Y. Frasconi P. Schmidhuber J. (2001). Gradient flow in recurrent nets: the difficulty of learning long-term dependencies, in A Field Guide to Dynamical Recurrent Neural Networks, eds Kremer S. C. Kolen J. F. (IEEE Press). Available online at: http://citeseerx.ist.psu.edu/viewdoc/summary?doi=10.1.1.24.7321

[B34] Hutchins J. B. Barger S. W. (1998). Why Neurons die: cell death in the nervous system. Anat. Rec. 253, 79–90. 9700393 10.1002/(SICI)1097-0185(199806)253:3<79::AID-AR4>3.0.CO;2-9

[B35] Jung M. W. McNaughton B. L. (1993). Spatial selectivity of unit activity in the hippocampal granular layer. Hippocampus 3, 165–182. 8353604 10.1002/hipo.450030209

[B36] Kali S. Dayan P. (2002). Replay, repair and consolidation, in Advances in Neural Information Processing Systems 15, eds Thrun S. Obermayer K. (Cambridge, MA: MIT Press), 19–26. 10.1007/s10856-014-5331-0

[B37] Karlsson M. P. Frank L. M. (2009). Awake replay of remote experiences in the Hippocampus. Nat. Neurosci. 12, 913–918. 10.1038/nn.2344 19525943 PMC2750914

[B38] LeCun Y. (1985). A learning scheme for asymmetric threshold networks, in Proceedings of Cognitiva 85 (Paris), 599–604.

[B39] Lehmann K. Butz M. Teuchert-Noodt G. (2005). Offer and demand: proliferation and survival of Neurons in the dentate gyrus. Eur. J. Neurosci. 21, 3205–3216. 10.1111/j.1460-9568.2005.04156.x 16026459

[B40] Luu P. Sill O. C. Gao L. Becker S. Wojtowicz J. M. Smith D. M. (2012). The role of adult hippocampal neurogenesis in reducing interference. Behav. Neurosci. 126, 381–391. 10.1037/a0028252 22642883 PMC3477487

[B41] Marin-Burgín A. Mongiat L. A. Pardi M. B. Schinder A. F. (2012). Unique processing during a period of high excitation/inhibition balance in adult-born Neurons. Science 335, 1238–1242. 10.1126/science.1214956 22282476 PMC3385415

[B42] Marr D. (1971). Simple memory: a theory for archicortex. Philos. Trans. R. Soc. Lond. B Biol. Sci. 262, 23–81. 4399412 10.1098/rstb.1971.0078

[B43] McAvoy K. Besnard A. Sahay A. (2015). Adult hippocampal neurogenesis and pattern separation in DG: a role for feedback inhibition in modulating sparseness to govern population-based coding. Front. Syst. Neurosci. 9:120. 10.3389/fnsys.2015.00120 26347621 PMC4542503

[B44] McClelland J. L. McNaughton B. L. O'Reilly R. C. (1995). Why there are complementary learning systems in the Hippocampus and neocortex: insights from the successes and failures of connectionist models of learning and memory. Psychol. Rev. 102, 419–457. 7624455 10.1037/0033-295X.102.3.419

[B45] McNaughton B. L. Morris R. G. M. (1987). Hippocampal synaptic enhancement and information storage within a distributed memory systems. Trends Neurosci. 10, 408–415.

[B46] Murdock B. (1962). The serial position effect of free recall. J. Exp. Psychol. 64, 482–488.

[B47] Myers C. E. Scharfman H. E. (2009). A role for hilar cells in pattern separation in the dentate gyrus: a computational approach. Hippocampus 19, 321–337. 10.1002/hipo.20516 18958849 PMC2723776

[B48] Myers C. E. Scharfman H. E. (2011). Pattern separation in the dentate gyrus: a role for the ca3 backprojection. Hippocampus 21, 1190–1215. 10.1002/hipo.20828 20683841 PMC2976779

[B49] Nair V. Hinton G. E. (2009). 3-D object reCognition with deep belief nets. Adv. Neural Inf. Process. Syst. 22, 1339–1347. Available online at: http://papers.nips.cc/paper/3872-3d-object-recognition-with-deep-belief-nets.pdf

[B50] O'Reilly R. C. McClelland J. L. (1994). Hippocampal conjunctive encoding, storage, and recall: avoiding a trade-off. Hippocampus 4, 661–682. 7704110 10.1002/hipo.450040605

[B51] Oswald A.-M. M. Reyes A. D. (2008). Maturation of intrinsic and synaptic properties of layer 2/3 pyramidal Neurons in mouse auditory cortex. J. Neurophysiol. 99, 2998–3008. 10.1152/jn.01160.2007 18417631 PMC3056441

[B52] Piatti V. C. Ewell L. A. Leutgeb J. K. (2013). Neurogenesis in the dentate gyrus: carrying the message or dictating the tone. Front. Neurosci. 7:50. 10.3389/fnins.2013.00050 23576950 PMC3616253

[B53] Rolls E. T. Treves A. (1998). Neural Networks and Brain Function. New York, NY: Oxford University Press.

[B54] Rolls E. T. (1987). Information representation, processing and storage in the brain: analysis at the single neuron level, in The Neural and Molecular Bases of Learning, eds Changeux J.-P. Konishi M. (Chichester: Wiley), 503–540.

[B55] Rumelhart D. E. Hinton G. E. Williams R. J. (1986). Learning internal representations by back-propagating errors. Nature 323, 533–536.

[B56] Salakhutdinov R. Hinton G. (2010). Replicated softmax: an undirected topic model, in Advances in Neural Information Processing Systems, eds Bengio Y. Schuurmans D. Lafferty J. D. Williams C. K. I. Culotta A. (Vancouver, CA: Curran Associates, Inc.), 2010.

[B57] Salakhutdinov R. Mnih A. Hinton G. (2007). Restricted boltzmann machines for collaborative filtering, in Machine Learning, Proceedings of the Twenty-fourth International Conference (ICML 2004), ACM (New York, NY; Corvalis, OR: AAAI Press), 791–798.

[B58] Schmidt-Hieber C. Jonas P. Bischofberger J. (2004). Enhanced synaptic plasticity in newly generated granule cells of the adult Hippocampus. Nature 429, 184–187. 10.1038/nature02553 15107864

[B59] Smolensky P. (1986). Information processing in dynamical systems: foundations of harmony theory, in *Parallel Distributed Processing* Vol. 1, *Chapter 6*, eds Rumelhart D. E. McClelland J. L. (Cambridge: MIT Press), 194–281.

[B60] Snyder J. S. Kee N. Wojtowicz J. M. (2001). Effects of adult neurogenesis on synaptic plasticity in the rat dentate gyrus. J. Neurophysiol. 85, 2423–2431. 11387388 10.1152/jn.2001.85.6.2423

[B61] Stocco A. Lebiere C. Anderson J. R. (2011). Conditional routing of information to the cortex: a model of the basal ganglia's role in cognitive coordination. Psychol. Rev. 117, 541–574. 10.1037/a0019077 20438237 PMC3064519

[B62] Temprana S. G. Mongiat L. A. Yang S. M. Trinchero M. F. Alvarez D. D. Kropff E.. (2015). Delayed coupling to feedback inhibition during a critical period for the integration of adult-born granule cells. Neuron 85, 116–130. 10.1016/j.neuron.2014.11.023 25533485 PMC4329739

[B63] Islama T. Fiebigb D. G. Meade N. (2002). Modelling multinational telecommunications demand with limited data. Int. J. Forecast. 18, 605–624. 10.1016/S0169-2070(02)00073-0

[B64] Treves A. Rolls E. T. (1992). Computational constraints suggest the need for two distinct input systems to the hippocampal ca3 network. Hippocampus 2, 189–200. 1308182 10.1002/hipo.450020209

[B65] Wang S. Scott B. W. Wojtowicz J. M. (2000). Heterogenous properties of dentate granule neurons in the adult rat. J. Neurobiol. 42, 248–257. 10.1002/(SICI)1097-4695(20000205)42:2<248::AID-NEU8>3.0.CO;2-J 10640331

[B66] Weisz V. I. Argibay P. F. (2009). A putative role for neurogenesis in neurocomputational terms: inferences from a hippocampal model. Cognition 112, 229–240. 10.1016/j.cognition.2009.05.001 19481201

[B67] Weisz V. I. Argibay P. F. (2012). Neurogenesis interferes with the retrieval of remote memories: forgetting in neurocomputational terms. Cognition 125, 13–25. 10.1016/j.cognition.2012.07.002 22841299

[B68] Winocur G. Becker S. Luu P. Rosenzweig S. Wojtowicz J. (2012). Adult hippocampal neurogenesis and memory interference. Behav. Brain Res. 277, 464–469. 10.1016/j.bbr.2011.05.032 21669236

[B69] Wiskott L. Rasch M. J. Kempermann G. (2006). A functional hypothesis for adult hippocampal neurogenesis: avoidance of catastrophic interference in the dentate gyrus. Hippocampus 16, 329–343. 10.1002/hipo.20167 16435309

[B70] Wojtowicz J. M. (2006). Irradiation as an experimental tool in studies of adult neurogenesis. Hippocampus 16, 261–266. 10.1002/hipo.20158 16435311

[B71] Zhao C. Teng E. M. Summers R. G. Jr. Ming G. L. Gage F. H. (2006). Distinct morphological stages of dentate granule Neuron maturation in the adult mouse Hippocampus. J. Neurosci. 26, 3–11. 10.1523/JNEUROSCI.3648-05.2006 16399667 PMC6674324

[B72] Zwietering M. H. Jongenburger I. Rombouts F. M. van't Riet K.. (1990). Modeling of the bacterial growth curve. Appl. Environ. Microbiol. 56, 1875–1881. 16348228 10.1128/aem.56.6.1875-1881.1990 PMC184525

